# Clinical efficacy observation of repetitive transcranial magnetic stimulation combined with auditory integration training in children with ASD

**DOI:** 10.3389/fpsyt.2026.1704732

**Published:** 2026-01-28

**Authors:** Qing-hong Hao, Jin-ying Wang, Jin-di Yang, Yao Tong, Lei Xiao, Xiao-qin Zeng, Wei Li, Su-fen Hu, Zhi-hai Lv

**Affiliations:** Department of Child Rehabilitation, Longgang District Maternity & Child Healthcare Hospital of Shenzhen City (Longgang Maternity and Child Institute of Shantou University Medical College), Shenzhen, China

**Keywords:** auditory integration training, autism spectrum disorder, combined intervention, randomized controlled trial, repetitive transcranial magnetic stimulation

## Abstract

**Background:**

Autism spectrum disorder (ASD) has become a major public health issue of global concern and has attracted the attention of researchers in recent years. Due to the high incidence and persistent lifelong dysfunction of children with ASD, exploring active and effective intervention strategies plays a key role in their prognosis. Repetitive transcranial magnetic stimulation (rTMS) and auditory integration training (AIT) are both promising neuromodulation methods and play an important role in clinical intervention in children with ASD. This study aims to explore the clinical effectiveness of rTMS and AIT combined intervention compared with single intervention through randomized controlled experiments, and provide scientific basis for clinical intervention in ASD.

**Methods:**

A total of 60 participants with ASD were randomly assigned to the study group (rTMS combined with AIT) and the control group (rTMS) in a 1:1 ratio, with 30 participants in each group. The outcome indicators were the Autism Behavior Checklist (ABC), the Childhood Autism Rating Scale (CARS), the Strengths and Difficulties Questionnaire (SDQ), and the Repetitive Behavior Scale-Revised (RBS-R). Clinical data were collected at baseline and after intervention. Data were processed and analyzed by SPSS.

**Results:**

After 12 weeks of intervention, the total scores of ABC, CARS, SDQ and RBS-R in the two groups were significantly improved compared with those before intervention (*P* < 0.05). Compared with the control group, after 12 weeks of intervention, the total scores of ABC and CARS, as well as the total scores and some dimensions of SDQ and RBS-R were significantly improved (*P* < 0.05).

**Discussion:**

The intervention therapy of rTMS combined with AIT has significant efficacy in children with ASD, which can significantly improve the core symptoms and emotional behavioral problems of children with ASD. This method is worthy of being promoted and applied in clinical practice.

**Clinical trial registration:**

https://www.chictr.org.cn/showproj.html?proj=225665, identifier ChiCTR2400082706.

## 1 Background

According to the Diagnostic and Statistical Manual of Mental Disorders, Fifth Edition (DSM-5), autism spectrum disorder (ASD) is a complex neurodevelopmental disorder characterized primarily by persistent deficits in social interaction and communication, along with restricted, repetitive patterns of behavior (1). Additionally, individuals with ASD often experience sensory processing abnormalities, emotional and behavioral challenges, and significant difficulties in self-care and social adaptation (2). According to the World Health Organization, approximately 1 in 100 children worldwide is diagnosed with ASD (3). Large-scale epidemiological studies in China indicate a prevalence rate of approximately 0.7% (4). Given its high prevalence and long-term impact on individual development (5), early assessment, diagnosis, and intervention for ASD are critically important. Currently, the specific pathogenesis of this disorder remains unclear, and no specific therapeutic drugs are available. Therefore, actively exploring effective clinical intervention methods has become a key focus in current research and practice.

Non-invasive brain stimulation (NIBS) techniques, such as repetitive transcranial magnetic stimulation (rTMS), have seen increasing application in ASD treatment in recent years and have been validated by numerous researchers (6, 7). By stimulating neural and vascular structures in specific brain regions, TMS can modulate cortical blood flow and promote neurotransmitter release, thereby helping regulate brain function in affected children (8). Current rTMS interventions for ASD predominantly target brain areas such as the dorsolateral prefrontal cortex (DLPFC), medial prefrontal cortex, and supplementary motor area (9). As a key region involved in emotional regulation, decision-making, attentional control, and adaptive behavior, DLPFC dysfunction is closely linked to core ASD symptoms such as social impairments and repetitive/stereotyped behaviors. Multiple studies indicated that bilateral low-frequency rTMS over the DLPFC significantly reduces repetitive behaviors in ASD patients (10–12). Additional studies reported significant improvements in social behaviors following low-frequency rTMS intervention in ASD (13, 14). These findings consistently suggested that DLPFC dysfunction represents a key neural mechanism underlying core ASD symptoms, particularly impairing social skills and behavioral regulation. With its excellent safety profile and accumulating evidence of efficacy, rTMS offers a promising intervention strategy for improving clinical symptoms in ASD patients.

Auditory Integration Training (AIT) is an audio intervention method developed by French physician Guy Bernard, designed to improve abnormal sound sensitivity in individuals, particularly those with neurodevelopmental disorders such as ASD (15, 16). AIT employs specialized equipment to filter sound signals by selectively amplifying or attenuating specific frequency bands. This stimulates auditory integration centers, promoting functional reorganization in the cerebral cortex regarding frequency perception and processing, thereby exerting therapeutic effects (17). Existing research indicated that AIT effectively alleviates core symptoms in children with ASD, including repetitive behaviors, emotional dysregulation, and communication difficulties (18). Yao’s study further demonstrated that this intervention significantly enhances children’s emotional stability, social interaction skills, and language comprehension levels (19). Furthermore, AIT is easily implemented in hospital or laboratory settings, facilitating researchers’ collection of physiological data during intervention to objectively evaluate treatment responses and mechanisms (20). Multiple studies supported AIT’s efficacy in improving auditory processing abilities in children with ASD, suggesting that enhanced sound perception and information processing may be closely linked to improvements in language comprehension, expression, and social communication functions (20, 21).

The combined application of rTMS and AIT in ASD treatment has demonstrated potential synergistic effects. rTMS promotes neuroplasticity by modulating the excitatory-inhibitory balance of cortical neurons (22); while AIT optimizes auditory information processing through frequency filtering techniques (20). Research indicated that combining these two interventions not only helped correct auditory processing abnormalities but also modulated cerebral oxygenation activity and neurotransmitter release. This results in multifaceted improvements in language cognition, emotional behavior, and social functioning among children with ASD (23, 24). From a neurobiological perspective, rTMS primarily targets the prefrontal cortex and associated executive control networks, while AIT focuses on auditory cortex and social cognition-related brain regions. Their combination enables broad regulation across multiple neural networks, potentially yielding more comprehensive therapeutic outcomes. Although preliminary evidence supported the positive effects of combined intervention, high-quality research in this field remains scarce (24, 25). Therefore, in-depth research is urgently needed to systematically evaluate the synergistic mechanisms and clinical efficacy of combining rTMS and AIT, providing more robust scientific evidence for evidence-based treatment of ASD.

This study employs a randomized parallel-group controlled trial design to investigate whether combined rTMS and AIT intervention yields greater improvements in core symptoms and emotional-behavioral issues compared to rTMS alone in children with ASD. Findings are expected to provide evidence-based support for optimizing clinical intervention strategies for ASD.

## 2 Methods

### 2.1 Design and setting

**Figure 1:**
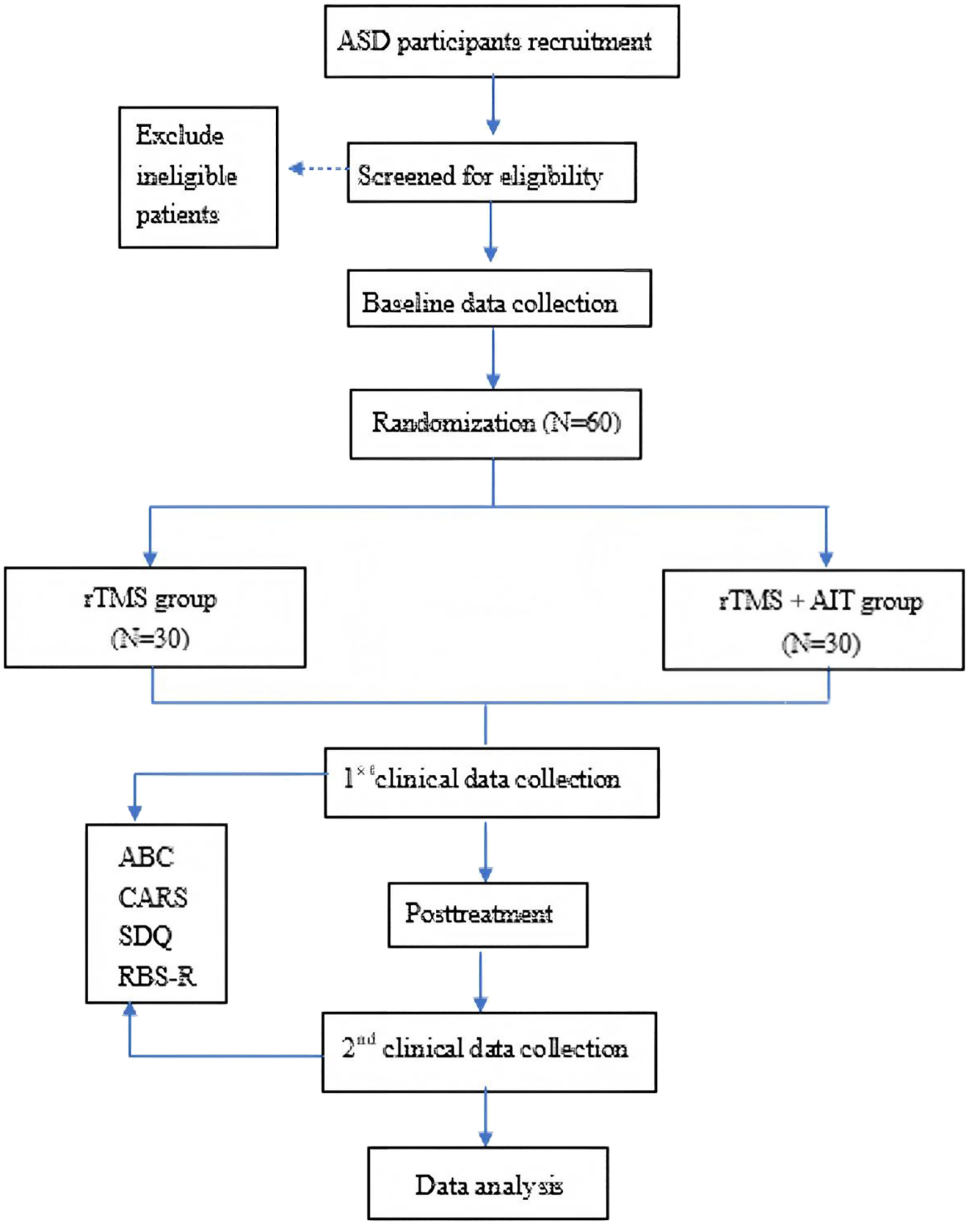
Flowchart of the trial.

**Figure 2:**
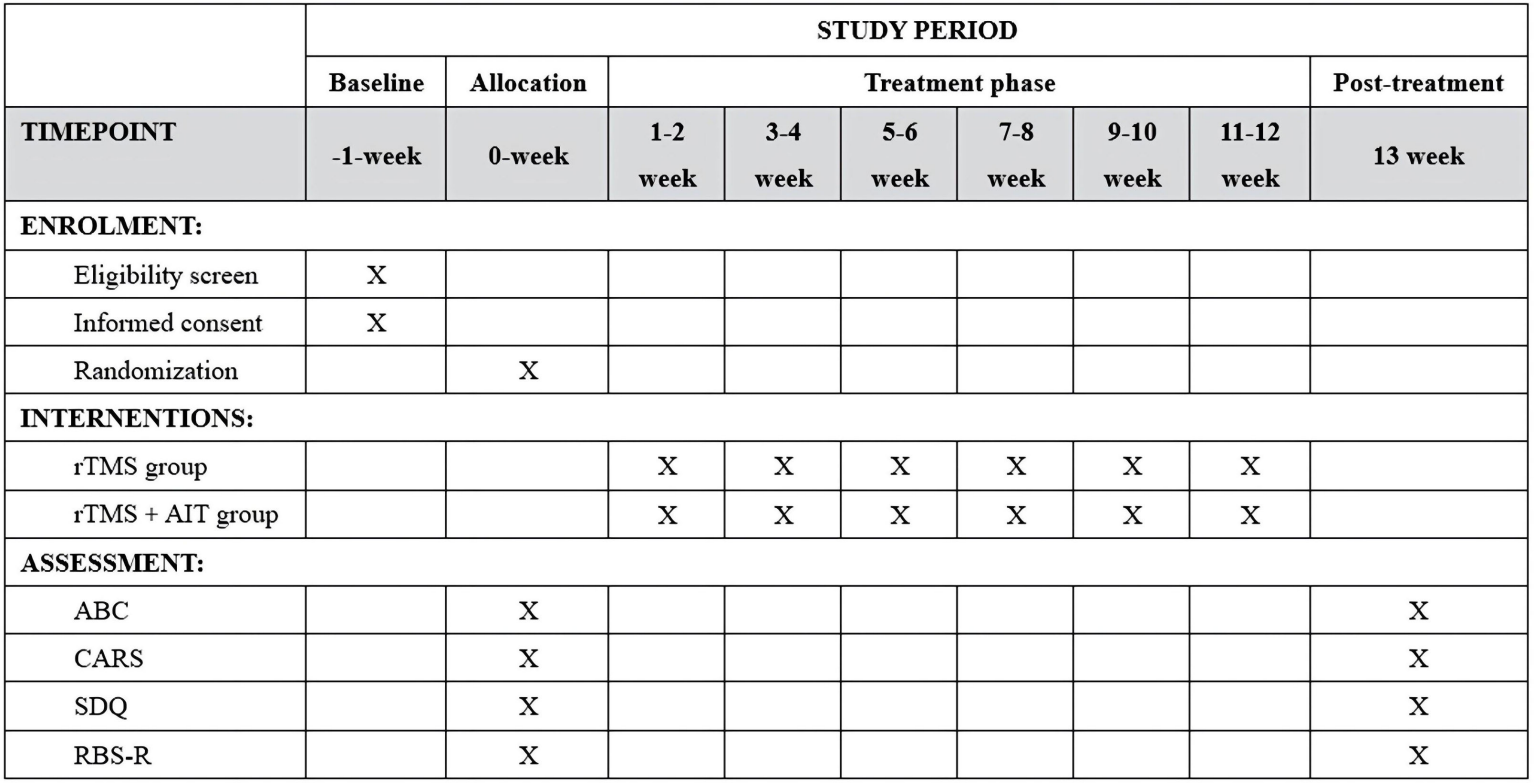
Trial schedule.

This was a randomized, parallel-group controlled trial conducted at the Shenzhen Longgang District Maternal and Child Health Hospital. Participants were randomly assigned using a random number table to either the study group (rTMS combined with AIT) or the control group (rTMS). Patients, evaluators, therapists, data analysts, and other researchers were unaware of the assignment. The trial design flowchart is shown in **Figure 1**, and participant timepoints is illustrated in **Figure 2**.

### 2.2 Participants

The participants of this study were inpatients and outpatients from the Children’s Rehabilitation Department of Shenzhen Longgang District Maternal and Child Health Hospital. The family members of patients who meet the inclusion criteria would be informed of the potential benefits and possible risks of this study. After the family members’ wishes were sought, those who agreed to be included in the experiment would sign the informed consent form.

### 2.3 Eligibility criteria

#### 2.3.1 Inclusion criteria

Patients who meet the following criteria would be included:

1. Meet the diagnostic criteria for ASD in the Diagnostic and Statistical Manual of Mental Disorders (5th edition) (26);
2. Aged 3–6 years old;
3. Routine EEG examination showed no obvious abnormalities: EEG screening was conducted in a standardized soundproof room using electrodes placed according to the international 10–20 system to record resting EEG activity in children while awake. The recordings were interpreted by a specialist physician (27);
4. The child’s sense of smell, vision, hearing and other indicators were normal, and there were no other physical and mental diseases;
5. The child’s guardian knew the content of this study and signed the informed consent form.

#### 2.3.2 Exclusion criteria

Patients with any of the following criteria would be excluded:

1. Children with obvious abnormalities in cranial MRI and severe cardiopulmonary insufficiency;
2. Participants exhibiting abnormal EEG findings, epileptiform discharges (e.g., spike-and-wave activity), or significant background activity abnormalities (e.g., focal slow waves) were excluded.
3. Children with schizophrenia, epilepsy, deaf-muteness, metabolic diseases or other mental illnesses;
4. Children who have recently taken medication or nutritional supplements to improve related symptoms;
5. Through clinical interviews with the parents, children with significant hearing loss were excluded;
6. There were metal foreign bodies in the brain or around the stimulation coil, pacemakers or implanted electronic devices;
7. Children who have received relevant interventions or comprehensive rehabilitation training in the past.

### 2.4 Informed consent process

Due to the particularity of the participants included in this study, the informed consent form would need to be signed by the parents or other guardians. The consent form would be signed before the start of the research procedure. At the same time, parents would be informed of the research process, precautions and confidentiality of personal information.

### 2.5 Sample size

At present, there is a lack of research on rTMS combined AIT therapy at home and abroad, so we referred to similar studies to estimate the sample size of this study. According to relevant literature (28, 29), α=0.05, test power 1-β=0.90, σ=6.5, two-sided test, and PASS 15 software was be used to calculate the sample size to be 15 cases per group. Considering a 20% dropout rate, this study plant to enroll 60 children with ASD, with 30 participants in each group.

### 2.6 Randomization and allocation concealment

A researcher (WL) who was not involved in patient recruitment and treatment used SPSS software (version 25.0) to randomly divide all participants into two groups, with 30 patients in each group. The sequence number, group and random number was placed in a kraft envelope, and each envelope was be numbered. If the participant met the inclusion and exclusion criteria, the envelope would be opened in the order of enrollment, and the grouping was completed according to the random number and grouping information in the envelope. Other researchers in this study were blind to the treatment allocation.

### 2.7 Intervention plan and grouping

Each group of participants received conventional comprehensive rehabilitation treatment, including speech and cognitive training, social training, occupational therapy, sensory integration training, music therapy (25, 30). 30 minutes/time, once/day, 5 times/week, 4 weeks as a course of treatment, a total of three courses of treatment.

#### 2.7.1 rTMS group

On the basis of conventional comprehensive rehabilitation treatment, the CCY-1 transcranial magnetic stimulator and the circular magnetic stimulation coil for children (Wuhan Yiruide Company) was used. The resting motor threshold (RMT) of the child was measured when the child undergone rTMS treatment for the first time. The measurement steps were as follows:

1. Wear the positioning cap correctly. The intersection of the naso-occipital line (the line connecting the root of the nose to the posterior occipital tuberosity) and the temporoparietal line (the line connecting the depressions at the ends of the two zygomatic arches) was the child’s Cz point. Make sure that the child’s Cz point coincides with the Cz point on the positioning cap.
2. Use the rTMS motor evoked potential special electrode line to record the motor evoked potential (MEP). The recording electrode is placed on the belly of the left-hand abductor pollicis brevis, the reference electrode is placed on the tendon at the distal end, and the ground electrode is placed on the wrist. The center of the circular coil is directly opposite the Cz point.
3. Use single pulse mode stimulation, stimulate 10 times, at least 5 of which can induce the movement of the abductor pollicis brevis muscle (evoked potential reaches 50μV or above), then the stimulation intensity is the resting motor threshold (Motor Threshold, MT) (31, 32).

During treatment, the child took a seat and wore a positioning cap, with the center of the coil aimed at the DLDFC. This study selected bilateral DLPFC for stimulation (stimulating the left DLPFC area in the first 6 weeks and the right DLPFC area in the last 6 weeks) (33). The frequency was set to 1Hz, the stimulation intensity was 80%RMT, 400 pulses each time, 20s interval between every 10 pulses, 5 times a week, and a total of 12 weeks as a course of treatment. In addition, due to the immature development of the cerebral cortex of children and the language and social disorders of ASD children themselves, their resting motor threshold was more likely to be undetectable. For children who cannot be detected, 30%-50% of the maximum output intensity of the instrument could be used for stimulation.

#### 2.7.2 rTMS combined with AIT group

On the basis of conventional rehabilitation treatment, two technologies, rTMS+AIT, were used for intervention. The therapist could reasonably arrange the time of the two treatments according to the subject’s time. Please note that due to equipment and time constraints, this study did not perform individual frequency adjustments based on audiometric measurements. However, at enrollment children with significant hearing impairment were excluded based on clinical interviews with their parents or guardians.

AIT intervention was carried out using Shenzhen Elite RT510 Auditory Integration Training Instrument. The intervention course was once a day, 30 minutes each time (listen to 1 treatment plan), 5 times/week, for a total of four weeks. The specific steps were (34):

1. Turn on the power of the main unit, turn on the headphone switch, and connect the treatment accessories to the main unit;
2. Select the treatment frequency, 2KHz and 8KHz for both left and right channels, adjust the intensity to 10, and select the corresponding age group and treatment plan according to the patient’s age (distinguishing between those over 4 years old and those under 4 years old);
3. Adjust the earphones (distinguish between the left and right sides) and put them on the child, then click start;
4. After the treatment, remove the treatment accessories from the child and turn off the power.

### 2.8 Outcome indicators

1. Autism Behavior Checklist (ABC) (35): This scale is completed by parents and is suitable for screening and assisting in the diagnosis of ASD patients aged 8 months to 28 years. It includes 57 behavioral symptoms of children with ASD, categorized into five factors: sensory abilities, communication abilities, motor skills, language, and self-care. Each item is scored on a scale of 1 to 4, with a total score ≥ 53 indicating a screening cutoff for ASD, and a total score ≥ 67 indicating a positive diagnosis for ASD.
2. Childhood Autism Rating Scale (CARS) (36): This scale is used for symptom assessment in children with ASD over 2 years old, comprising 15 items. Qualified personnel will conduct the assessments, with each item scored from 1 to 4. A total score of 30-36, with fewer than 5 items scoring below 3, indicates mild to moderate ASD; a total score > 36 with at least 5 items scoring above 3 indicates severe ASD.
3. Strengths and Difficulties Questionnaire (SDQ) (37): Developed by Dr. Goodman et al., this questionnaire assesses emotional and behavioral problems in children aged 2-17, and is also suitable for measuring emotional and behavioral issues in special children, such as those with ASD. The SDQ consists of 25 scoring items divided into four areas of difficulty: emotional symptoms, conduct problems, hyperactivity, and peer relationship problems, along with one prosocial behavior scale. Parents rate their child’s emotional and behavioral performance over the past six months on a Likert scale: “0” means not true, “1” means somewhat true, and “2” means certainly true. Higher scores on the difficulty scales indicate more severe emotional and behavioral problems, while higher scores on the prosocial behavior scale indicate better prosocial behavior. The Chinese version of the SDQ has good reliability and validity.
4. Repetitive Behavior Scale-Revised (RBS-R) (38): This scale comprises six core components: stereotyped behavior, self-injurious behavior, compulsive behavior, ritualistic behavior, fixated behavior, and restricted behavior, totaling 43 items. A higher total score indicates more stereotyped behaviors. The Chinese version of this scale has good reliability and validity and can serve as an effective assessment tool for evaluating the treatment effects on stereotyped behaviors in children with ASD.

### 2.9 Statistical analysis

Statistical analysis was performed using SPSS 25.0 software. For continuous variables meeting normal distribution and homogeneity of variance, data were expressed as mean ± standard deviation ( $\bar{\text{x}}$ ± s). Comparisons between groups were conducted using the independent samples t-test, while comparisons within groups before and after treatment employed the paired samples t-test. Nonparametric tests were used for continuous variables that did not meet normal distribution. Categorical data were analyzed using the chi-square test. *P* < 0.05 was set to indicate statistically significant differences.

### 2.10 Safety

Case report forms (CRFs) monitored and recorded adverse events (AEs) throughout the trial. No serious AEs attributable to the intervention have been observed to date.

## 3 Result

### 3.1 Participant characteristics

A total of 60 participants were finally included in this study, with 30 in the study group and 30 in the control group. There was no significant difference in age (Z=-1.199, P > 0.05) and gender (χ^2^ = 0.800, P > 0.05) between the two groups of participants, and the baseline was consistent. See **Table 1**.

**Table 1:** Study characteristic.

Baseline	Study group (M/F)	Control group (M/F)	Statistical value	P-value
Gender	24/6	21/9	χ^2^ = 0.800	0.371
Age	3.90 ± 0.85	4.25 ± 0.967	Z = -1.199	0.231
ABC	51.70 ± 12.46	54.70 ± 14.44	t=-0.703	0.486
CARS	35.90 ± 5.15	35.90 ± 5.15	t=-0.027	0.989

M, Male; F, Female.

### 3.1 Comparison of ABC and CARS scores between the two groups before and after intervention

Before intervention, there was no significant statistical difference in ABC (t=-0.703, *P* > 0.05) and CARS (t=-0.027, *P* > 0.05) scores between the study group and the control group. After 12 weeks of intervention, both groups showed a decrease in ABC and CARS scores, with statistically significant differences (*P* < 0.05). Compared with the control group, the study group demonstrated significantly better ABC (t=-2.307, P = 0.027) and CARS (Z=-3.034, P = 0.002) scores after 12 weeks of intervention. See **Table 2**.

**Table 2:** Comparison of ABC and CARS scores between the two groups of children before and after intervention.

Group	ABC		CARS	
	Pre	Post	Pre	Post
study group (n=30)	51.70 ± 12.46	30.25 ± 12.57^a^	35.90 ± 5.15	21.30 ± 7.71^a^
control group (n=30)	54.70 ± 14.44	38.55 ± 10.04^a^	35.90 ± 5.15	27.65 ± 8.15^a^
statistical value	t=-0.703	t=-2.307	t=-0.027	Z=-3.034
P-value	0.486	0.027^b^	0.989	0.002^b^

Compared with pre-intervention within the group, *^a^p* < 0.05; Compared with the control group, the difference in the study group was more significant, *^b^p* < 0.05. Pre: pre-intervention; Post: post-intervention

### 3.2 Comparison of SDQ scores between the two groups before and after intervention

Before intervention, there were no statistically significant differences in SDQ total scores (t=0.738, *P* > 0.05) or scores across all domains between the intervention group and the control group (*P* > 0.05). After 12 weeks of intervention, statistically significant differences were observed between the two groups in SDQ total scores and scores for hyperactivity, peer relationship problems, and prosocial behavior (*P* < 0.05). Compared with the control group, the intervention group demonstrated significantly better outcomes in SDQ total scores (t=-2.654, P = 0.012), emotional symptoms (Z=-2.600, P = 0.011), conduct problems (Z=-2.435, P = 0.021), and prosocial behavior (t=2.329, P = 0.025) after 12 weeks of intervention (*P* < 0.05). See **Table 3**.

**Table 3:** Comparison of SDQ scores before and after intervention.

Group	Emotion		Conduct issues		Hyperactivity		Peer interaction		Prosocial behavior		Total	
	Pre	Post	Pre	Post	Pre	Post	Pre	Post	Pre	Post	Pre	Post
study group (n=30)	2.55 ± 1.50	1.00 ± 1.03	2.10 ± 0.91	1.05 ± 0.83	6.30 ± 1.81	3.75 ± 1.33 ^a^	5.50 ± 1.82	2.95 ± 1.57 ^a^	2.90 ± 2.81	4.85 ± 2.16 ^a^	19.35 ± 3.50	13.65 ± 2.41 ^a^
control group (n=30)	2.50 ± 2.09	2.10 ± 1.37	1.90 ± 1.02	1.85 ± 1.04	6.20 ± 2.10	4.85 ± 1.69 ^a^	5.55 ± 1.61	3.95 ± 1.15 ^a^	2.20 ± 2.09	3.40 ± 176 ^a^	18.35 ± 4.95	16.15 ± 3.45 ^a^
statistical value	t=0.087	Z=-2.600	t=-0.650	Z=-2.435	t =0.162	Z–1.889	t=-0.092	t=-2.299	Z=-0.631	t=2.329	t=0.738	t=-2.654
P-value	0.931	0.011 ^b^	0.547	0.021 ^b^	0.872	0.068	0.543	0.119	0.547	0.025 ^b^	0.465	0.012 ^b^

Compared with pre-intervention within the group, *^a^p* < 0.05; Compared with the control group, the difference in the study group was more significant, *^b^p* < 0.05.

### 3.3 Comparison of RBS-R scores between the two groups before and after intervention

Before intervention, there were no statistically significant differences in the total RBS-R scores (t=0.553, *P* > 0.05) or scores across all dimensions between the study group and the control group (*P* > 0.05). After 12 weeks of intervention, statistically significant differences were observed between the two groups in both the total RBS-R score and scores across all subscales (*P* < 0.05). Compared with the control group, the intervention group demonstrated significantly lower total RBS-R scores (Z=-3.432, P = 0.000) and scores for stereotyped behaviors (t=-4,154, P = 0.000), compulsive behaviors(Z=-3.197, P = 0.001), ritualistic behaviors (Z=-3.675, P = 0.000), and restricted behaviors (Z = 2.317, P = 0.023) after 12 weeks of intervention. See **Table 4**.

**Table 4:** Comparison of RBS-R scores before and after intervention.

Group	Stereotyped behavior		Self-harm		Compulsive behavior		Ritual behavior		Monotonous behavior		Restricted activities		Total	
	Pre	Post	Pre	Post	Pre	Post	Pre	Post	Pre	Post	Pre	Post	Pre	Post
study group (n=30)	5.20 ± 2.12	1.85 ± 0.93 ^a^	8.20 ± 1.54	3.45 ± 2.70 ^a^	9.30 ± 2.03	2.30 ± 2.06 ^a^	7.65 ± 2.28	1.10 ± 1.29 ^a^	11.35 ± 2.87	9.35 ± 4.39 ^a^	10.10 ± 4.27	3.85 ± 2.85 ^a^	46.30 ± 5.70	14.70 ± 5.90 ^a^
control group (n=30)	5.90 ± 1.94	3.80 ± 1.89 ^a^	8.75 ± 1.97	2.35 ± 1.60 ^a^	8.25 ± 2.53	4.30 ± 1.56 ^a^	5.85 ± 2.78	3.50 ± 2.12 ^a^	11.60 ± 3.63	8.75 ± 4.19 ^a^	8.85 ± 4.39	4.15 ± 2.06 ^a^	45.15 ± 7.36	22.75 ± 7.62 ^a^
statistical value	Z=-1.089	t=-4.154	t=-0.983	Z=1.566	t=-1.448	Z=-3.197	Z=-1.671	Z=-3.675	Z=-0.746	Z=-1.604	Z=-0.455	Z=-2.317	t=0.553	Z=-3.432
P-value	0.283	0.000 ^b^	0.332	0.126	0.156	0.001 ^b^	0.102	0.000 ^b^	0.461	0.117	0.659	0.023 ^b^	0.584	0.000 ^b^

Compared with pre-intervention within the group, *^a^p* < 0.05; Compared with the control group, the difference in the study group was more significant, *^b^p* < 0.05.

## 4 Discussion

This study investigated the clinical efficacy of combined rTMS and AIT therapy for children with ASD through a randomized parallel-group controlled trial. Results demonstrated that after 12 weeks of intervention, the combined treatment group (intervention group) showed significantly greater improvement than the rTMS-only group (control group) across multiple dimensions, including core symptoms, behavioral and emotional problems, and repetitive and stereotyped behaviors. The following sections provide an in-depth discussion of the study’s key findings.

First, the most significant finding of this study is that the combined intervention of rTMS and AIT produced a synergistic effect. As shown in the results, although both groups exhibited reduced ABC and CARS scores post-intervention compared to baseline, the decrease was significantly greater in the combined treatment group than in the control group. Current research indicates that while studies on rTMS combined with AIT treatment remain limited, existing investigations have demonstrated the superior efficacy of rTMS in combination with other interventions-such as EEG biofeedback and acupuncture-compared to monotherapy (39, 40). ABC and CARS are commonly used screening and diagnostic scales for assessing core symptoms of ASD and are considered authoritative instruments (41). This result indicates that the combined intervention not only effectively alleviates core ASD symptoms but also yields superior outcomes compared to rTMS stimulation alone. We hypothesize that rTMS may improve children’s neural functional basis by modulating cortical excitability and neural network connectivity in brain regions such as the prefrontal and temporal lobes, thereby further enhancing clinical features (42). AIT, targeting the widespread auditory processing abnormalities in children with ASD, enhances auditory filtering and sensory integration abilities through training. This reduces anxiety, distractibility, and abnormal behaviors triggered by abnormal sensory input (16, 43). The combination of these approaches forms a closed-loop “neurostimulation-behavioral training” intervention. By simultaneously addressing the core deficits of ASD at both the brain neural network and sensory processing system levels, it achieves superior clinical efficacy.

Secondly, combined therapy also demonstrated significant advantages in improving comorbid symptoms and emotional-behavioral issues. SDQ results indicated more pronounced improvements in the study group across dimensions such as emotional symptoms, conduct problems, and prosocial behavior. This suggests that combined intervention not only targets core symptoms but may also stabilize emotions by reducing sensory overload in children with ASD, thereby decreasing behavioral problems and increasing the likelihood of prosocial behavior (44). Gao (45) employed the SDQ to evaluate the efficacy of rTMS intervention for ASD, yielding results consistent with our study. Both investigations confirmed that neuromodulation techniques demonstrate significant advantages in enhancing emotional regulation, behavioral control, and social skills. Another study applied AIT, integrating behavioral observations with psychophysiological and neurophysiological indicators to explore the abnormal neural mechanisms and functional basis underlying sound processing in individuals with autism. Results showed that following Berard AIT treatment, EEG patterns in children with ASD exhibited positive trends, with reduced differences compared to typically developing children, indicating a normalization of neural responses (20). Concurrently, parent assessment questionnaires clearly indicated significant improvements across multiple behavioral domains, including irritability, hyperactivity, and repetitive behaviors. Similarly, on the RBS-R scale, the study group demonstrated significant advantages across multiple dimensions including stereotyped behaviors, compulsive behaviors, and restricted behaviors. This further confirms the program’s effectiveness in alleviating the characteristic, restricted behavioral patterns observed in children with ASD. Repetitive stereotyped behaviors are often associated with abnormalities in the basal ganglia circuitry of the brain, and research indicates that rTMS can modulate the activity of these circuits (10). Another study indicated that rTMS can improve repetitive and stereotyped behaviors in autism by stimulating the DLPFC cortical target. For instance, Barth et al. (46) administered low-frequency rTMS to the bilateral DLPFC of children with autism over 12 weeks of treatment (once weekly). Repetitive and stereotyped behaviors were quantified using the RBS-R scale. Results demonstrated significant improvement in these behaviors following the intervention. Research indicates that seeking sensory stimulation is one cause of repetitive behaviors in children with ASD, and perceptual training yields both immediate and sustained effects in addressing these behaviors (47). AIT specifically improves children’s sensory processing systems, reducing their sensory sensitivity. This allows children to no longer require repetitive behaviors to seek sensory stimulation or escape discomfort. The synergistic effects of both interventions collectively contributed to positive changes in emotional and behavioral issues.

The clinical significance of this study lies in providing a novel, highly effective multimodal intervention approach for ASD rehabilitation. Traditional single-modality treatments often exhibit certain limitations. This research demonstrates that the organic integration of neuromodulation technology with sensory training can promote more comprehensive and profound improvements across multiple functional domains in children with ASD. This provides robust empirical evidence for developing more effective integrated intervention protocols.

### 4.1 Study limitations

This study also has several limitations. First, while rigorous behavioral assessments are used, neurophysiological indicators (e.g., EEG, fNIRS) are not included. Therefore, this study cannot directly reveal the underlying neural mechanisms of the combined intervention. Future studies should verify these mechanisms in conjunction with neuroimaging techniques. Second, this study did not collect the initial cognitive and adaptive function data of the subjects. In the subsequent research, this information will be supplemented. Third, the ADOS or ADI-R were not used as standardized diagnostic tools in this study. Future research will incorporate standardized diagnostic tools to enhance diagnostic consistency. Then, In this study, the AIT intervention did not include detailed individualized frequency adjustments (such as frequency filtering in Berard AIT), resulting in a lack of personalized treatment. Future research will integrate audiometric measurements to optimize individualized intervention through tailored AIT parameters. Finally, the lack of long-term follow-up data means the long-term efficacy of the combined treatment remains unclear.

In summary, the combined treatment of rTMS and AIT represents an effective intervention strategy superior to rTMS alone, significantly improving core symptoms, emotional-behavioral issues, and repetitive stereotyped behaviors in children with ASD. Future research should expand sample sizes and utilize neuroimaging techniques such as functional magnetic resonance imaging (fMRI) and electroencephalography (EEG) to explore the underlying brain mechanisms of this combined approach. This will lay the foundation for achieving personalized, precision rehabilitation treatment for ASD.

## Data Availability

The raw data supporting the conclusions of this article will be made available by the authors, without undue reservation.
